# The HEX 110 Hexamerin Is a Cytoplasmic and Nucleolar Protein in the Ovaries of *Apis mellifera*

**DOI:** 10.1371/journal.pone.0151035

**Published:** 2016-03-08

**Authors:** Juliana Ramos Martins, Márcia Maria Gentile Bitondi

**Affiliations:** 1 Departamento de Genética, Faculdade de Medicina de Ribeirão Preto, Universidade de São Paulo, Monte Alegre, Ribeirão Preto, São Paulo, Brazil; 2 Departamento de Biologia, Faculdade de Filosofia, Ciências e Letras de Ribeirão Preto, Universidade de São Paulo, Monte Alegre, Ribeirão Preto, São Paulo, Brazil; Brunel University, UNITED KINGDOM

## Abstract

Hexamerins are insect storage proteins abundantly secreted by the larval fat body into the haemolymph. The canonical role of hexamerins consists of serving as an amino acid reserve for development toward the adult stage. However, in *Apis mellifera*, immunofluorescence assays coupled to confocal laser-scanning microscopy, and high-throughput sequencing, have recently shown the presence of hexamerins in other organs than the fat body. These findings have led us to study these proteins with the expectation of uncovering additional functions in insect development. We show here that a honeybee hexamerin, HEX 110, localizes in the cytoplasm and nucleus of ovarian cells. In the nucleus of somatic and germline cells, HEX 110 colocalized with a nucleolar protein, fibrillarin, suggesting a structural or even regulatory function in the nucleolus. RNase A provoked the loss of HEX 110 signals in the ovarioles, indicating that the subcellular localization depends on RNA. This was reinforced by incubating ovaries with pyronin Y, a RNA-specific dye. Together, the colocalization with fibrillarin and pyronin Y, and the sensitivity to RNase, highlight unprecedented roles for HEX110 in the nucleolus, the nuclear structure harbouring the gene cluster involved in ribosomal RNA production. However, the similar patterns of HEX 110 foci distribution in the active and inactive ovaries of queens and workers preclude its association with the functional status of these organs.

## Introduction

Through the larval stage, the fat body of holometabolous insects synthesizes and secretes a large quantity of proteins. These larval storage proteins that increase considerably in the hemolymph up to the last larval instar have been mostly identified as hexamerins because they are generally composed by six polypeptide subunits. With few exceptions, these proteins with molecular weights in the range of 5 x 10^5^, progressively disappear from the hemolymph during the quiescent pupal stage, when the insect does not feed [1]. The hypothesis that hexamerins are a source of amino acids for the insect development was confirmed by using radioactively labeled purified calliphorin, the larval storage protein of *Calliphora vicina*. After injection of the labeled protein in larvae, the radioactivity was found in adult tissues, as it would be expected if calliphorin had been recycled for adult tissue formation [2]. In *Drosophila*, comparable proteins were named larval storage proteins (Lsp) [3, 4], and this specific nomenclature has been maintained so far. During metamorphosis, the storage proteins are sequestered from the hemolymph by the fat body where they are compacted in protein granules for further recycling. This was first demonstrated in *Calpodes ethlius* [5], but it is a general occurrence among insects [6].

Currently, it is known that hexamerins are hemocyanin evolutionary-derived proteins that have lost the ability to bind copper ions and transport oxygen [7–9]. At the molecular structure level, the members of the hexamerin protein family can be recognized by the presence of the conserved C (PF03723.5), N (PF03722.5) and M (PF00372.10) hemocyanin domains (Pfam database) [10].

HEX 110 is one of the four hexamerins in the honeybee, *Apis mellifera*. It is encoded by the *hex 110* gene, which is present as a single copy in the genome and is separated from the other three hexamerin genes, which are tandemly arrayed in the Group 8.21 of the honeybee genome assembly (version 4.5). *hex 110* is positioned in the Group 11.18. [11, 12]. HEX 110 is composed of subunits in the 110 kDa range as determined by SDS-PAGE, and its N-terminus was identified by amino acid sequencing [13].

HEX 110 has a molecular mass higher than that typically exhibited by hexamerin subunits in general (in the range of 70 kDa) and displays a high content (20.9%) of glutamine/glutamic acid residues [12]. These features are also displayed by hexamerins from other hymenopterans, viz, the parasitic wasp *Nasonia vitripennis* [14], other wasp species [15] and ants [16], and also by a member of the hemocyanin-derived family in *Drosophila*, the FBP1 hexamerin-receptor [17]. The other hexamerins described to date do not have these features.

HEX 110 proteins are abundantly concentrated in the haemolymph of late larval, pupal and early pharate adult stages, decaying afterward to basal levels. Although at lower levels, the fat body, the main site of hexamerin biosynthesis, displays a similar HEX 110 developmental profile [18]. Since HEX 110 is massively synthesized by the larval fat body and extensively secreted and stored in the haemolymph, it fits the criteria for inclusion in the class of insect storage proteins. The fact that HEX 110 molecules are progressively depleted from the haemolymph as metamorphosis proceeds is consistent with its putative role in attending the amino acid demand for pharate adult development, culminating in the ecdysis of the adult bee. Similar behaviour and function were proposed for the HEX 110 potential orthologs in ants [16] and wasps [15].

Using a specific antibody in experiments of immunolocalization and confocal laser-scanning microscopy, we previously investigated the subcellular localization of HEX 110 in the honeybee fat body during the metamorphic apex, i.e., the larval-pupal transition. HEX 110 was localized in the cytoplasm and nucleus of the fat body cells [18]. In the trophocytes, the cytoplasmic HEX 110 foci were visualized as dense particles of different sizes, matching in shape and localization the protein granules identified much earlier through microscopy studies in the fat body of the honeybee [19–21] and *C*. *ethlius* [5], which apparently are formed by the fusion of vesicles containing endocytosed haemolymph proteins. In the honeybee, such protein granules had been observed shortly before pupation, increased in quantity during the early pupal stage and then disappeared near the time of adult ecdysis [19–21]. The developmental profile of HEX 110 in the hemolymph and its presence in fat body protein granules [18] are consistent with a function as a genuine storage protein, which is recycled for adult tissues formation.

However, the presence of HEX 110 in the fat body cell nuclei of honeybees metamorphosing from larvae into pupae [18] suggests that it plays other roles in addition to being a storage protein. Supporting our findings on HEX 110’s nuclear localization, Begna *et al*. [22] detected HEX 110 in the nuclear proteome of worker and queen honeybee larvae in the fourth and fifth instars using nuclear protein enrichment, two-dimensional electrophoresis and mass spectrometry. Moreover, tackling the view that hexamerins are exclusively larval storage proteins, *hex 110* transcripts were also detected in the fat body of adult worker bees, in the developing gonads of workers and drones, and in the ovaries of egg-laying queens, as demonstrated through RT-PCR [12]. Whole transcriptome sequencing using RNA-seq also identified *hex 110* transcripts during the adult stage of the honeybee [23] and *Apis cerana* cerana [24]. In addition, the HEX 110 protein was detected in the brain of adult worker bees through multidimensional protein identification technology [25].

The expression of *hex 110* in other honeybee organs, besides the fat body, adds new questions on the roles of this protein. In this scenario, the ovaries appear as the relevant organs to be investigated, considering that caste-specific differences related to ovary structure and function are central issues in the theme of insect sociality evolution. Honeybee workers exhibit a marked reduction in the number of ovarioles, the structural units of the ovaries, in comparison to queens. In addition, in regular conditions, i.e., in colonies headed by a queen, oocyte growth is in general inhibited in the remaining worker ovarioles. Only exceptionally, in case of queen loss, the workers are released from the inhibitory effect of queen pheromone [26], and can then lay haploid, unfertilized eggs that will give rise to drones [27].

In this context we aimed to search for alternative roles of HEX 110. The current study describes its subcellular localization in the inactive and active ovarioles of workers and queens using a specific antibody and confocal laser-scanning microscopy. Fibrillarin, a nucleolar marker, was used in colocalization experiments. RNase A and pyronin Y, a dye that shows specificity towards RNAs, provided insights into RNA-dependent HEX 110’s subcellular localization.

## Materials and Methods

### Honeybees

Honeybee workers and queens (*Apis mellifera*, Africanized) were collected from hives maintained with standard beekeeping practices at the experimental apiary of the Department of Genetics, Faculty of Medicine, University of São Paulo, Ribeirão Preto, SP, Brazil.

### Anti-HEX 110 specificity

A custom-made polyclonal anti-HEX 110 was produced in rabbits (Rheabiotech, Campinas, SP, Brazil) using a stretch of amino acid residues (NLYTKYHGQYP) of the HEX 110 subunit (GenBank accession number ABU92559.1) predicted from the fully sequenced cDNA [12]. The specificity of the antibody was validated by using the fat body in an immunoprecipitation assay with anti-HEX 110, followed by identification of the precipitated proteins by mass spectrometry. Samples of fat body tissue dissected from 5^th^ instar larvae (staged according to Michelette and Soares [28]) were pooled, washed in PBS_1_ (137 mM NaCl, 2.7 mM KCl, 10 mM Na_2_HPO_4_, 1.7 mM KH_2_PO_4_, pH 7.4) and resuspended in 10 mM Tris-HCl, pH 7.5, 10 mM KCl, 5 mM MgCl_2_ and 10% protease inhibitor cocktail (Roche Applied Science). The tissue was lysed using an insulin syringe and centrifuged at 1000 x g for 5 min at 4°C to remove large cell debris and nuclei. The supernatant was transferred to a fresh tube and mixed with 1% Triton X-100 and 10.2 mL PBS_1_. Proteins were precipitated with 30.7 mL acetone for 16 h at 4°C. After centrifugation at 10,000 x g for 30 min at 4°C, the supernatant was discarded and the pellet was air-dried for 10 min at low temperature, resuspended in 2 mL of extraction buffer (Dynabeads Co-Immunoprecipitation Kit–Invitrogen), and centrifuged at 10,000 x g for 5 min at room temperature. The supernatant was transferred to a fresh tube and immediately used for immunoprecipitation with anti-HEX 110 and Dynabeads Antibody Coupling Kit (Invitrogen). Mass spectrometry was used to identify immunoprecipitated proteins and to check antibody specificity. Briefly, the immunoprecipitated sample was resuspended and run on SDS-PAGE based on Laemmli [29]. After electrophoresis, the gels were stained with colloidal Coomassie blue [30], or used for western blot with anti-HEX 110. The 110 kDa band identified in the Coomassie blue-stained gels was cut off and bleached with 50% acetonitrile for 1h, washed in 50 mM NH_4_HCO_3_ and in 100% acetonitrile for 10 min each. The gel piece was dried (Savant SC110 SpeedVac) for 10 min, digested with 10 μL of a trypsin solution (20 μg/mL trypsin, Proteomics Grade, Sigma Aldrich) in 50 mM ammonium bicarbonate during 30 min on ice, and incubated overnight at 37°C with 100 μL of 50 mM ammonium bicarbonate. The reaction was stopped by adding 1 μL of formic acid. The trypsin-digested peptides were extracted from the gel piece by incubating it in a mixture of 40 μL trifluoroacetic acid 2.5% (v/v) and acetonitrile 50% (v/v) for 3h. The trypsin-digested peptides were mixed with 18 μL of MALDI matrix (5 mg/mL α-cyano-4-hydroxycinnamic acid, 0.1% formic acid and 55% acetonitrile) and 1 μL was spotted on the MALDI target plates. MS and MS/MS spectra were obtained by using a MALDI-TOF/TOF UltrafleXtrememass spectrometer (BrukerDaltonics). The results were analyzed using Mascot software, assuming P<0.05for the search of proteins in the NCBI Database (www.ncbi.nlm.nih.gov) [31].

The immunoprecipitated samples were also used for western blot with anti-HEX 110. Following SDS-PAGE, the proteins were transferred to a nitrocellulose membrane (ImmunBlot™ PVDF Membrane, Bio-Rad). Non-specific binding sites were blocked by incubating the membrane for 16 h in 10% non-fat dried milk in PBS_2_ (50 mM Tris, 80 mM NaCl, 2 mM CaCl_2_, pH 8.5). HEX 110 was visualized by incubating the membrane for 1 h at room temperature with anti-HEX 110 diluted 1:1,000 in 10% non-fat dried milk in PBS_2_. The membrane was washed thoroughly in 0.05% Tween 20 in PBS_1_ and subsequently incubated for 1 h in a horseradish peroxidase labeled anti-rabbit IgG secondary antibody (GE Healthcare) diluted 1:12,000 in 0.05% Tween 20 in PBS_1_. After washing in 0.05% Tween 20 in PBS_1,_ the detection was carried out by using the ECL System (ECL™ Western Blotting Analysis System, GE Healthcare).

Antibody specificity was also validated through the use of the partially purified native HEX 110 as sample in PAGE followed by western blot. Haemolymph from fifth instar larvae was collected from a small lateral incision in the abdomen using glass microcapillaries. After mixing it with a protease inhibitors cocktail (Roche Applied Science), the haemolymph was diluted at the proportion of 5:1 (v/v) in an anticoagulant solution (182 mM KCl, 46 mM NaCl, 20 mM EDTA, 10 mM Tris, pH 7.2–7.4) containing crystals of phenylthiourea and centrifuged at 4°C for 5 min (at 3,000 x g, at 5,000 x g, and then for four times at 10,000 x g). The supernatant was ultracentrifuged in a 5–40% sucrose gradient at 75,000 x g for 15h at 4°C in a Sorvall WX-90 Ultracentrifuge (Thermo Scientific). One of the recovered fractions was used as a sample for HEX 110 molecular weight determination by native PAGE, which was carried out at 15–20 mA and 4°C using 3–12% polyacrylamide gradient. Following electrophoresis, the proteins were stained with 1% Coomassie Blue R-250 in ethanol, distillated water and acetic acid (5:5:1 v/v) for band visualization, or were transferred to the nitrocellulose membrane. Western blot was performed with anti-HEX 110 as described above.

Haemolymph (0.2 μL) supernatant (prepared as described above) was also used as samples in SDS-PAGE followed by western blots (as above). The membranes were revealed with anti-HEX 110 or with preimmune serum as a negative control for non-specific binding or cross-reactivity.

### HEX 110 immunolocalization in the ovarioles and colocalization with fibrillarin and RNA

Ovarioles of newly-emerged and nurse bee workers from queenright colonies, workers from a queenless colony and egg-laying queens were used for immunolocalization of HEX 110. After dissection in PBS_1_, the ovarioles were separated from each other and the peritoneal sheath involving each one was carefully removed. The ovarioles were fixed with 4% paraformaldehyde in PBS_1_ for 20 min and then classified into five stages (S0, S1, S2, S3 and S4) according to development degree. Fixed ovarioles were permeabilized with 0.1% Triton X-100 in PBS_1_ (TPBS) for 5 min (three washes), blocked with 1% BSA in 0.1% TPBS (TPBSA) for 5 min (five washes) and incubated in 10% normal goat serum (Invitrogen) in TPBSA for 1 h. The ovarioles were then incubated with anti-HEX 110 at a concentration of 1:50 (v/v) or with this antibody and anti-fibrillarin (Life Technologies) at a concentration of 1:400 (v/v) in 10% normal goat serum in TPBSA for 16h at 4°C. This was followed by three washes of 5 min and five washes of 20 min in TPBSA and incubation in 10% normal goat serum in TPBSA for 1h. Alexa Fluor 488 conjugated goat anti-rabbit antibody and Alexa Fluor 594 conjugated goat anti-mouse antibody (Life Technologies, 1:200 v/v dilution in TPBSA containing 10% normal goat serum) were added to the preparations, which were then incubated for 2 h at room temperature. After three washes of 5 min and five washes of 20 min in TPBSA, the ovarioles were incubated with a 1:4000 v/v dilution of DAPI in TPBS for 5 min and rinsed five times in TPBS for 5 min or incubated for 15 min in 1 μg propidium iodide (Invitrogen) diluted in 500 μL of 0.6% Triton X-100 in PBS_1_ and rinsed five times in TPBS.

Before mounting on slides, some ovarioles from queenright nurse workers were soaked for 20 sec in a 0.165 mM pyronin Y solution in PBS_1_. Pyronin Y (Sigma-Aldrich) is an intercalating cationic dye that shows specificity towards RNA [32, 33]. After washing 5 times in PBS_1_ (5 min each washing), the ovarioles were mounted on glycerol 80% (Merck) and examined under a Leica TCS-SP5 confocal microscope (Leica Microsystems). Some ovarioles were not incubated with primary antibodies (negative controls).

Colocalization between HEX 110 and fibrillarin or RNA was analyzed by selecting regions of interest (ROI) in the confocal images of the ovarioles. We used Fiji, an image-processing package based on ImageJ software (US National Institute of Health, Bethesda, MD, USA) to determine the colocalization of fluorescence signals (pixels) from two channels (red and green) in confocal images. Images were subjected to an automatic area thresholding (colocalization threshold). The degree of overlap, or colocalization, between pixels visualized through the two channels was estimated using Manders coefficient values M1 and M2, which are expressed in a range from 0 to 1 (0 means no overlap and 1 means total overlap) [34]. In our case, the M1 coefficient represents the degree of red signal overlapping the green signal, and the M2 coefficient represents the degree of green signal overlapping the red. The fluorescence intensity (%) was also calculated for the red and green channels.

### RNase treatment

RNase treatment was based on Chamousset *et al*. [35]. Ovaries from queenright nurse bees were dissected in PBS_1_. Ovarioles were rinsed with PBS_1_ and permeabilized by incubating them in PBS_1_ plus 0.1% Triton X-100 for 5 min at room temperature. Then they were incubated with RNase A (100 μg/ml; Thermo Scientific) in PBS_1_ for 20 min at room temperature. Controls were incubated only with PBS_1_. The ovarioles were rinsed three times with PBS_1_ and fixed with 4% paraformaldehyde in PBS_1_ for 20 min. This was followed by incubation with the specific antibody for HEX 110 immunolocalization and examination under a confocal microscope as specified above. The green fluorescence originated from HEX 110/Alexa-Fluor 488 labeling was quantified in selected areas of the confocal images taken from the RNase A-treated and untreated ovarioles by converting the images to 8 bits and quantifying the intensity of gray pixels. The values obtained were expressed as Mean (the sum of the gray values of the pixels in the figure area divided by the number of pixels), Min/Max (minimum/maximal gray value), and IntDen (integrated density, i.e., the product of area and mean gray value).

### *In silico* analyses

Potential RNA-binding sites were searched in the HEX 110 sequence using RNABindR, BindN, PPRint, SVM and RNABindRPlus softwares [36–40]. We selected the most significant motifs, and only the alignments passing a threshold of 80% were considered as significant. The cNLS Mapper tool (http://nls-mapper.iab.keio.ac.jp/cgi-bin/NLS_Mapper_form.cgi [41]) and the NetNES 1.1 Server [42] were used for predictions of nuclear localization signal (NLS) and nuclear export signal (NES) in the HEX 110 sequence. The web server Nuc-PLoc (http://chou.med.harvard.edu/bioinf/Nuc-PLoc) [43] was used for predicting HEX 110 subnuclear localization.

## Results

### Anti-HEX 110 specificity

The results shown in the current work are based on immunofluorescence microscopy, a method generally used to assess the localization of proteins of interest in cells and tissues. As the application of this method is mainly based on the specificity of the primary antibody, we first searched for evidence of specificity of the custom-made anti-HEX 110 through the use of immunoprecipitation, mass spectrometry and western blot.

**Fig 1:**
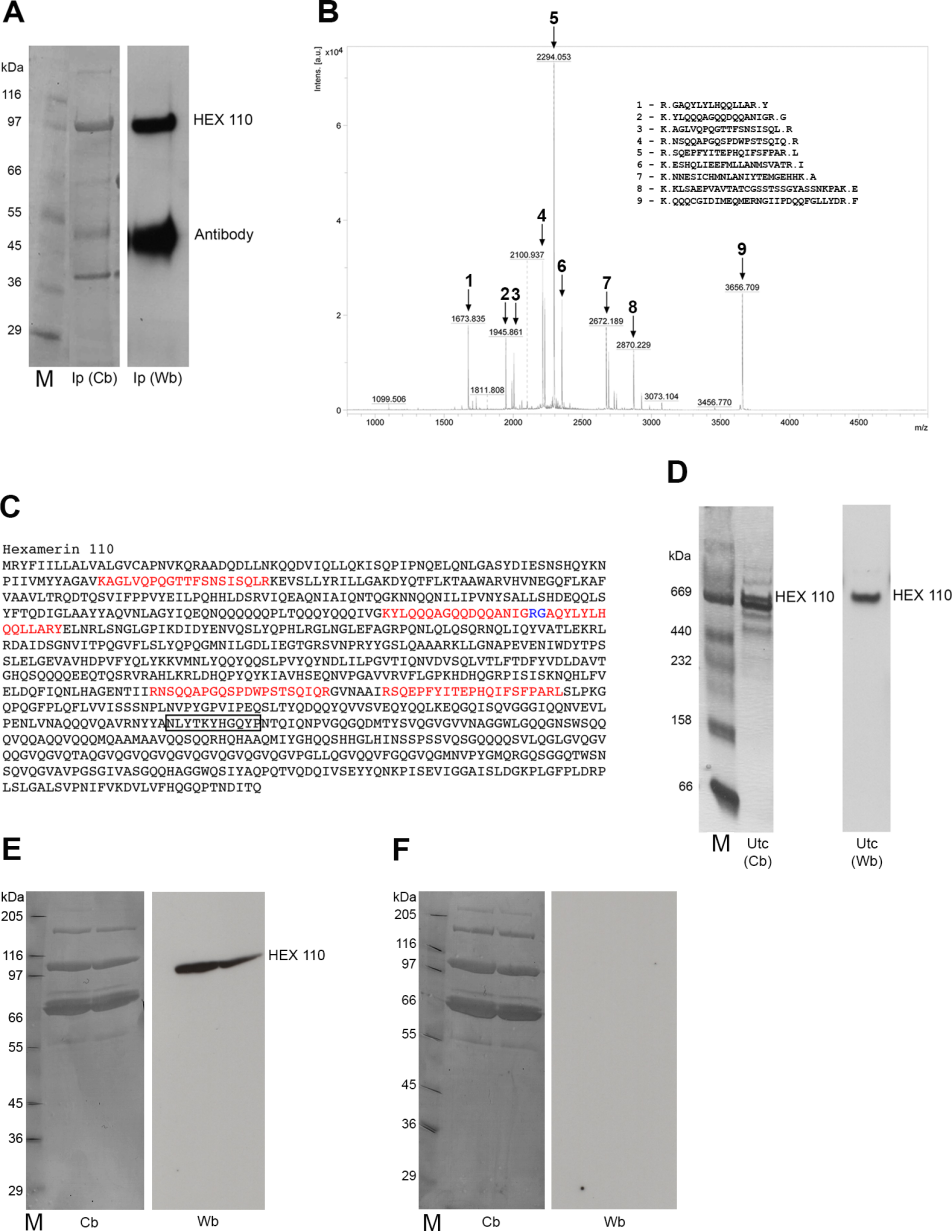
Specificity of the anti-HEX 110 antibody. (A) Larval fat body extracts (source of hexamerins) were incubated with the specific antibody for HEX 110 immunoprecipitation. The immunoprecipitate (Ip) was used as samples in SDS-PAGE stained with Coomassie blue (Cb) and western blot (Wb). The HEX 110 band position is indicated (the 45 kDa band is the anti-HEX 110 antibody present in the Ip). (B) Mass spectrogram of the Coomassie blue-stained HEX 110 band shown in A. The peaks 1 to 5 correspond to HEX 110 amino acid stretches (listed at the right); peaks 6–9 do not correspond to HEX 110 polypeptides. (C) The HEX 110 amino acid sequence as deduced from the fully sequenced cDNA [2] (GenBank accession number ABU92559). The five amino acid stretches identified by mass spectrometry are marked in red (overlapped amino acids are shown in blue). The amino acid sequence used for anti-HEX 110 synthesis (epitope) is marked by a rectangle. (D) Native PAGE stained with Coomassie blue (Cb) and western blot (Wb) of a partially purified fraction of larval haemolymph (source of hexamerins) obtained by ultracentrifugation (Utc). The western blot using the anti-HEX 110 antibody shows a band migrating at the 669 kDa position, thus suggesting the homohexameric nature of the native HEX 110 and confirming the antibody specificity. (E) SDS-PAGE stained with Coomassie blue (Cb) and western blot (Wb) using total haemolymph. The western blot revealed with anti-HEX 110 antibody shows specifically the HEX 110 subunit band. (F) SDS-PAGE stained with Coomassie blue (Cb) and western blot (Wb) using total haemolymph. The western blot was revealed with pre-immune serum as a negative control. M = molecular mass marker.

The antibody was produced from a HEX 110 amino acid stretch that has no match with other honeybee hexamerin sequences, or any other known honeybee protein, as verified by searching genome databases in BLAST analysis. The incubation of homogenized larval fat body (the main source of hexamerins) with this anti-HEX 110 antibody generated a pellet, which was assayed by Coomassie blue-stained SDS-PAGE and western blot (Fig 1A). A subunit band near the 110 kDa position was revealed in the Coomassie blue-stained gel and cross-reacted with anti-HEX 110. The Coomassie-blue stained 110 kDa band (as shown in Fig 1A) was cut out of the gel and trypsin-digested, and the resulting polypeptides were identified by mass spectrometry. Five peaks in the spectrogram (Fig 1B) matched stretches of the HEX 110 amino acid sequence (Fig 1C) that had been previously deduced by fully sequencing the cDNA (GenBank accession number ABU92559 [12]). These results show that the antibody was efficient in precipitating HEX 110. The peak 6 in the spectrogram resulted in a significant database hit, and matched a predicted honeybee DIS3-like exonuclease (exoribonuclease, GB41692). The peaks 7–9 corresponded to peptides without significant database hits and were assumed as contaminants. Alignment scores for HEX 110 polypeptide hits and for the DIS3-like exonuclease hit are given in S1 Table.

The specificity of the antibody was also demonstrated through native-PAGE and western blot analyses of a partially purified fraction of larval haemolymph obtained by ultracentrifugation in a 5–40% sucrose gradient (Fig 1D). The Coomassie blue-stained native gel revealed a band at the 669 kDa position, near the expected 660 kDa for the native protein, if the protein is considered a homohexamer made of 110 kDa subunits. The 669 kDa band cross-reacted with anti-HEX 110.

Since only a single band (~110 kDa) cross-reacted with anti-HEX 110 in western blots of haemolymph samples run on denaturing SDS-PAGE (Fig 1E), we could also infer that HEX 110 is comprised of polypeptide subunits of identical molecular mass. Aliquots of such haemolymph samples were also loaded on SDS-polyacrylamide gels subsequently processed for western blot using the pre-immune serum as a negative control (Fig 1F).

### HEX 110 in the inactive ovaries of queenright workers

**Fig 2:**
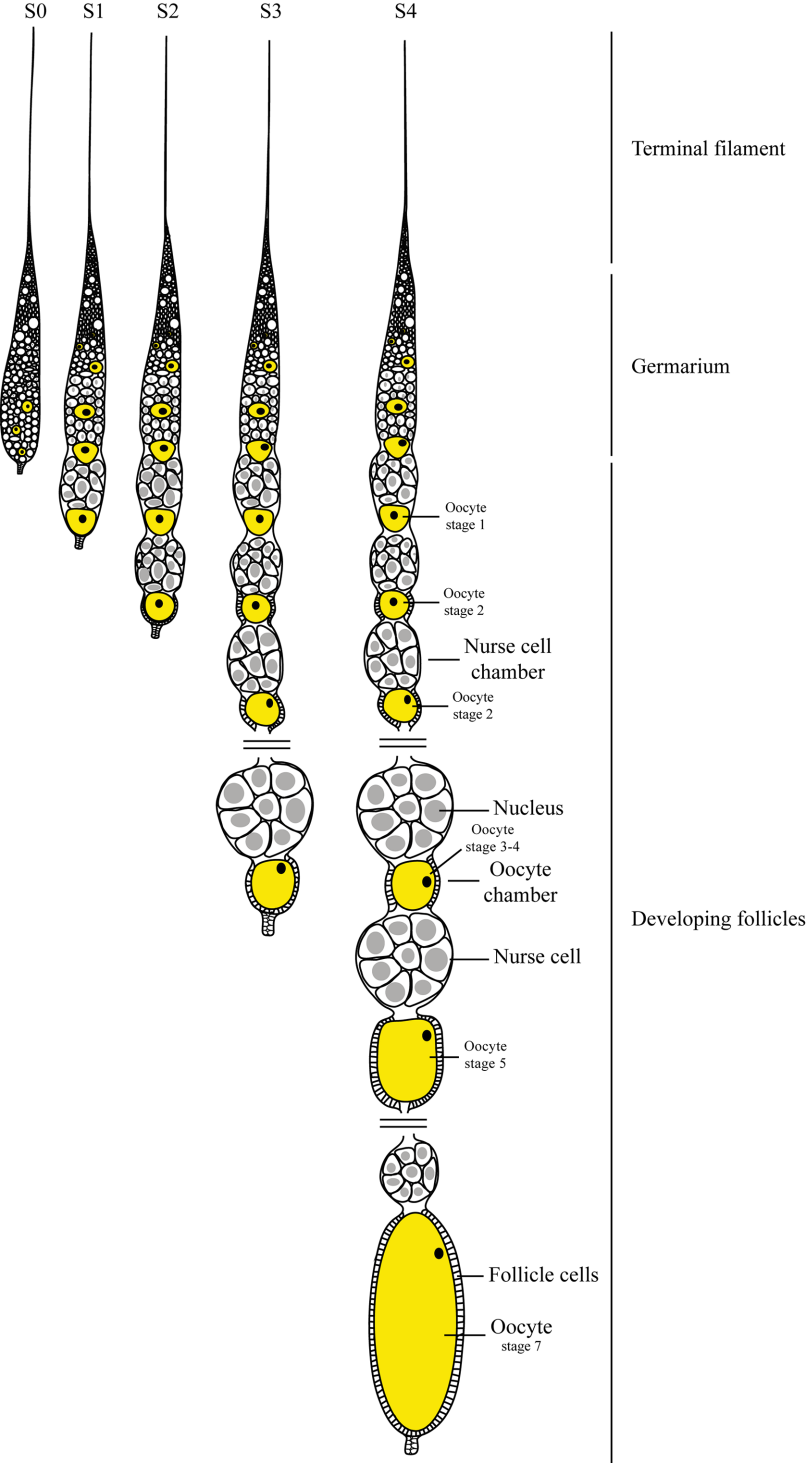
Ovariole structure and stages characterization. The ovariole comprises the anterior terminal filament, germarium and developing follicles (vitellarium) as indicated at the right. The oocytes are marked in yellow. The ovarioles are categorized according to the degree of development in stages S0, S1, S2, S3 and S4. Stage S0: presence of initial oocytes (cystocytes) and precursors of nurse and somatic cells in the germarium. Stage S1: an oocyte accompanied by differentiated nurse cells is positioned at the basal region of the ovariole. Stage S2: a slight constriction between the basal oocyte and accompanying nurse cells marks the beginning of oocyte chamber and nurse cell chamber separation; the basal oocyte is surrounded by follicle cells. Stage S3: a sequence of developing oocytes and nurse cell chambers is evident in the ovariole; the larger oocyte at the basal region is well-separated from the nurse cell chamber. Stage S4: presence of several developing oocytes and nurse cell chambers through the ovariole; a quasi-fully or a fully developed oocyte is evident at the most basal region. The classification of the maturing oocytes in stages 1 to 7 (stage 6 is not shown) was based in Wilson *et al*. [44].

**Fig 3:**
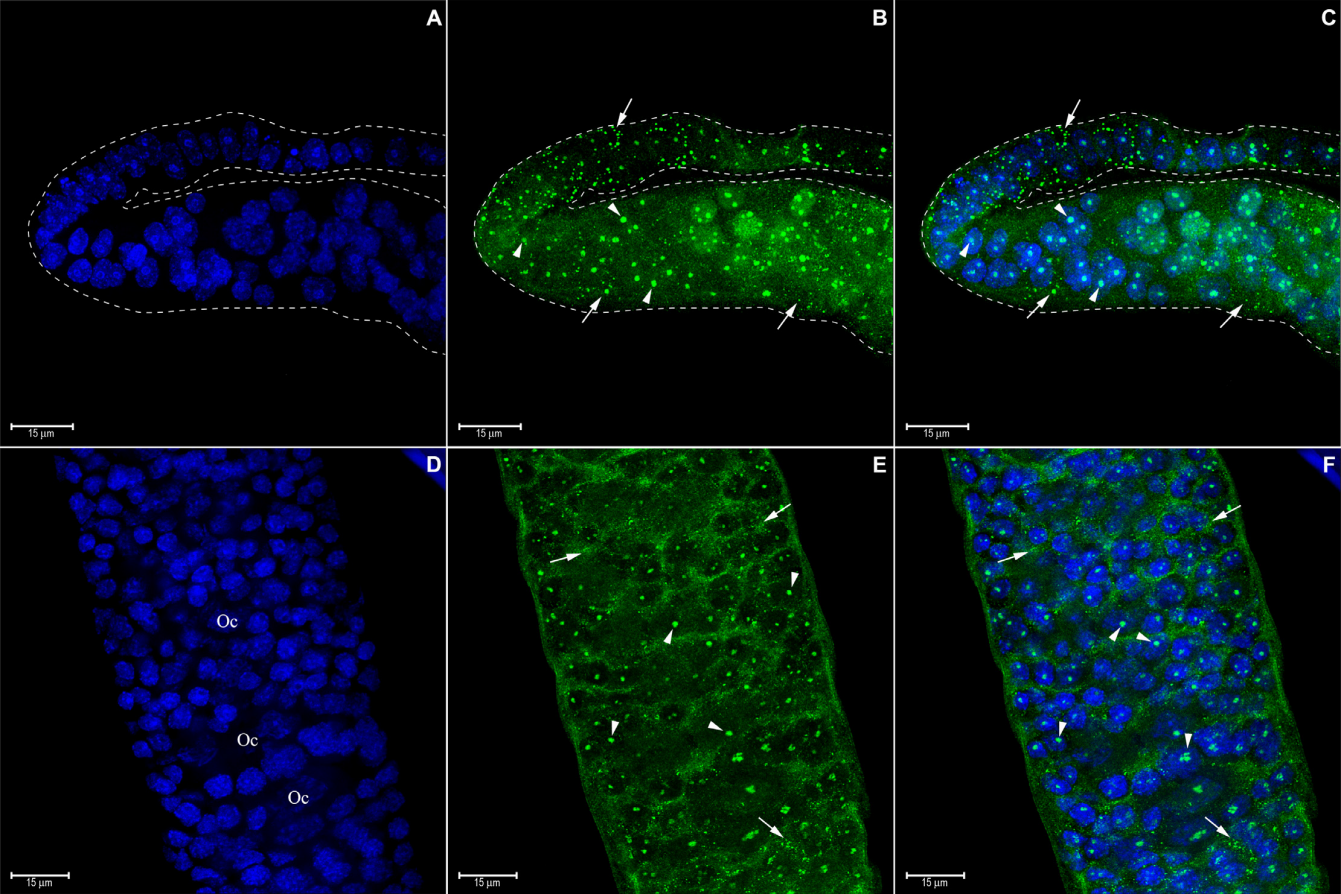
Immunolocalization of HEX 110 in the inactive ovarioles (stage S0) of queenright newly-emerged workers. (A-C) Part of the terminal filament (at the top) and upper region of the germarium (at the bottom). The dashed lines highlight the shape of the ovariole that appears folded in the images. (D-F) Lower region of the germarium. (A and D) DAPI-stained cell nuclei (blue). Initial oocytes (cystocytes) (Oc) are identified in D. (B and E) HEX 110 foci (green) in the cytoplasm (arrows) and nucleus (arrowheads) of germarium cells evidenced with anti-HEX 110/Alexa-Fluor 488. (C, F) Merged images: arrows and arrowheads point to the same cytoplasmic and nuclear HEX 110 foci seen in B and E.

HEX 110 localization was investigated in the slender and inactive ovaries of worker bees living in the presence of a queen (queenright workers), as well as in the active ovaries of queenless workers and queens. As inactive and active ovaries differ in ovariole length and morphology, and as a support for the description of HEX 110 immunolocalization patterns, we represented in Fig 2 the structure of the honeybee ovarioles and classified them into five successive stages (S0, S1, S2, S3 and S4). The maturing oocytes were classified in stages 1 to 7 according to Wilson *et al*. [44]. In the inactive ovarioles (stage S0, see Fig 2) of queenright newly emerged workers, HEX 110 was localized in the terminal filament and uppermost region of the germarium (Fig 3A–3C), and also in its lower region (Fig 3D–3F). Foci were seen in the cytoplasm and nucleus of ovariolar cells. The nucleoli appeared as the nuclear structures targeted by HEX 110.

**Fig 4:**
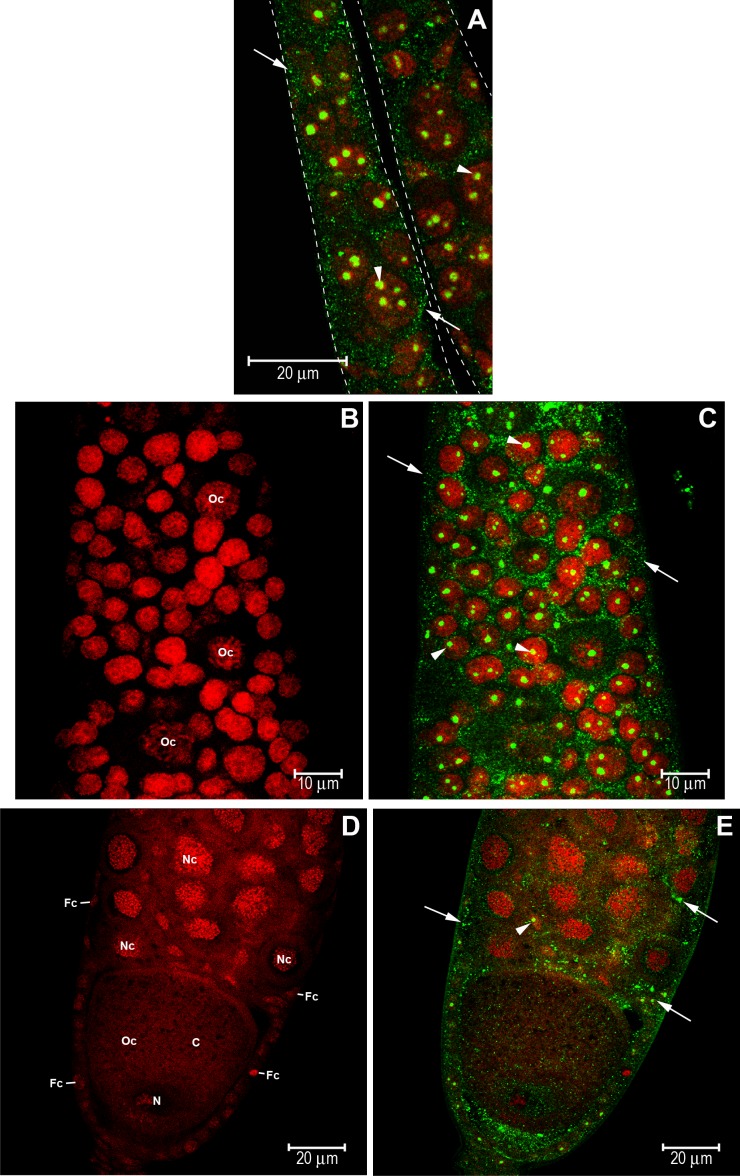
Immunolocalization of HEX 110 in ovarioles (stage S1) of nurse workers from a queenright colony. HEX 110 foci (green) were detected with anti-HEX 110/Alexa-Fluor 488. Propidium iodide (red) stains DNA and RNA and was used to highlight the nuclei. (A) Upper region of the germarium of two ovarioles (separated by dashed lines) showing germ- and somatic cell precursors containing cytoplasmic (arrows) and nuclear (arrowheads) HEX 110 foci. (B) Lower region of the germarium showing initial oocytes (cystocytes) (Oc) and nurse- and follicle cell precursors, these still morphologically undistinguishable from each other. (C) The same germarium region showing cytoplasmic (arrows) and nuclear (arrowheads) HEX 110 foci. (D) A follicle at the basal region of the ovariole showing a developing oocyte (Oc, stage 1, see Fig 2), and nurse cells (Nc). Oocyte nucleus (N) and cytoplasm (c) are indicated. Small follicle cells (Fc) are seen around the oocyte and nurse cell chamber. (E) The same follicle shown in D. Arrows point to cytoplasmic HEX 110 foci. The arrowhead points to nuclear HEX 110 foci in a follicle cell within the nurse cell chamber.

S1 staged ovarioles (see Fig 2) of queenright nurse bees also showed clear anti-HEX 110 labeling. HEX 110 foci were cytoplasmic and nuclear and were localized in the upper region of the germarium (Fig 4A), in the initial oocytes (cystocytes), and also in the precursors of nurse and somatic cells (Fig 4B and 4C) of the lower region of the germarium. A developing follicle formed by an oocyte (stage 1, see Fig 2) and nurse cells was observed in the basal region of the ovariole (Fig 4D and 4E). At this stage of follicle development (Fig 4E), the nurse cells and the oocyte did not show the conspicuous HEX 110 nuclear spot clearly observed in the precursor cells (Fig 4C). However, a follicle cell interspersed between the nurse cells (Fig 4E, arrowhead) showed HEX 110 nuclear labeling.

### HEX 110 in the active ovaries of queenless workers and queens and colocalization with the nucleolar protein, fibrillarin

**Fig 5:**
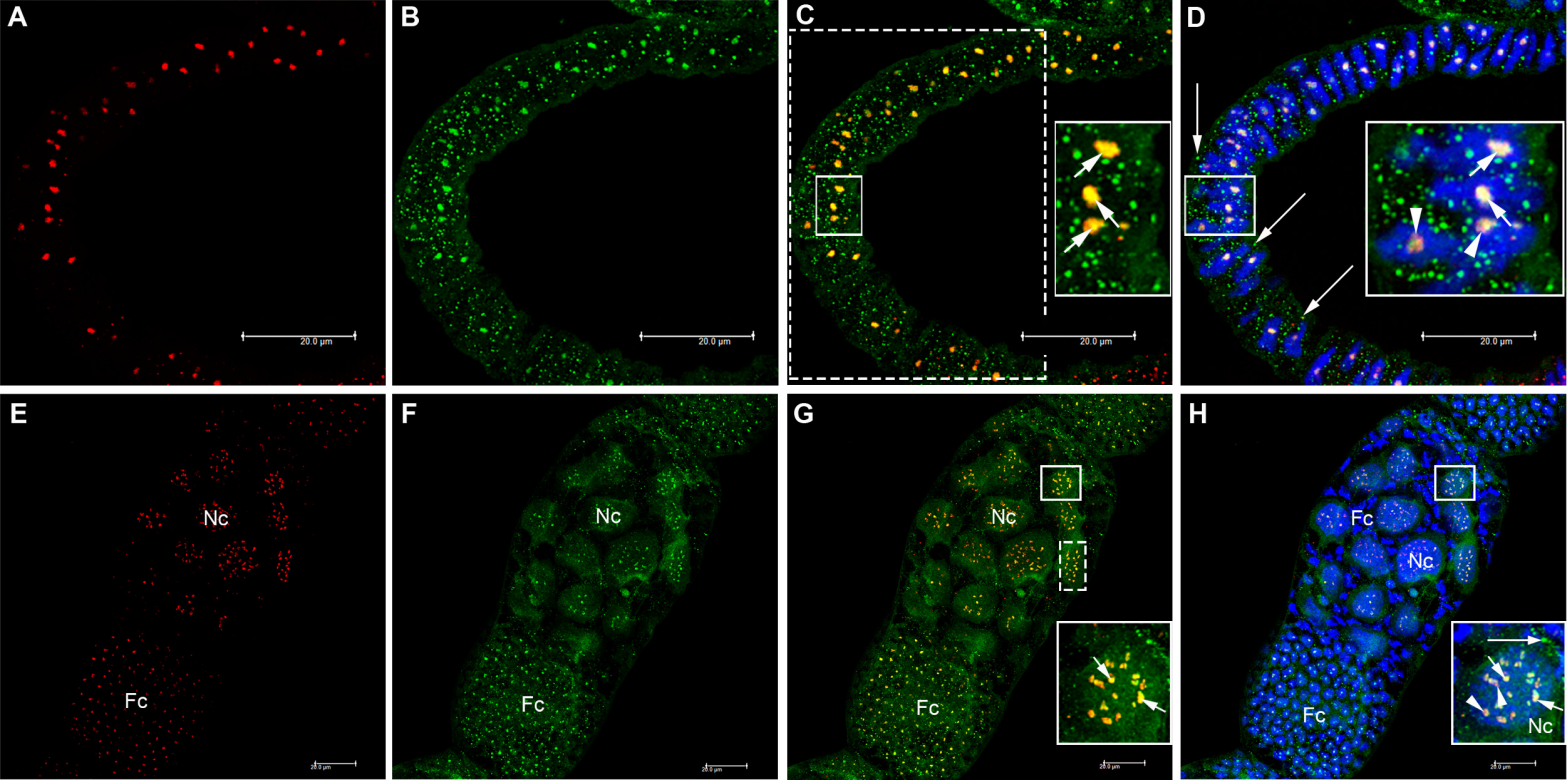
Colocalization of HEX 110 and fibrillarin, a nucleolar protein marker, in the active ovarioles (stage S3) of queenless workers. (A) Terminal filament of an ovariole stained with anti-Fibrillarin/Alexa-Fluor 594 (red foci). (B) The same image showing HEX 110 foci (green) identified with anti-HEX 110/Alexa-Fluor 488. (C) The merged image: anti-Fibrillarin/Alexa-Fluor 594 (red foci) and anti-HEX 110/Alexa-Fluor 488 (green foci). The insert highlights costained HEX 110/fibrillarin (yellow foci pointed by arrows). The area delimited by the dashed rectangle was used for estimation of Manders colocalization coefficients, M1 (red) and M2 (green), estimated as 0.3814 and 0.3462, respectively (red and green signals amounted to 37.45% and 27.11% of the selected area, respectively). (D) The same image added with DAPI-labeled nuclei (blue). The longer arrows point to HEX 110 foci in the cell cytoplasm. The insert highlights costained HEX 110/fibrillarin (yellow foci pointed by shorter arrows) and costained nucleolar DNA/fibrillarin (pink foci pointed by arrowheads). (E) A developing follicle stained with anti-Fibrillarin/Alexa-Fluor 594 (red foci) shows multiple nucleoli in the nurse cells (Nc) and a single nucleolus in the follicle cells (Fc) surrounding the oocyte (stage 2, see Fig 2) at the bottom of the figure. (F) The same image showing HEX 110 foci (green) identified with anti-HEX 110/Alexa-Fluor 488. (G) The merged image: anti-Fibrillarin/Alexa-Fluor 594 (red foci), anti-HEX 110/Alexa-Fluor 488 (green foci). The insert (nurse cell) highlights costained HEX 110/fibrillarin (yellow foci pointed by arrows). The area delimited by the dashed rectangle was used for estimation of Manders colocalization coefficients, M1 (red) and M2 (green), resulting in values of 0.6423 and 0.2203, respectively (red and green signals amounted to 79.83% and 28.36% of the delimited area, respectively). (H) The same image added with DAPI-labeled nuclei (blue). The insert (nurse cell) highlights costained HEX 110/fibrillarin (yellowish foci pointed by short arrows) and costained nucleolar DNA/fibrillarin (pink foci pointed by arrowheads). The longer arrow in this insert points to HEX 110 foci (green) in the cytoplasm of a nurse cell.

We then investigated the localization of HEX 110 in the active ovaries (S3 staged ovarioles, see Fig 2) of worker bees that were maintained for 19 days in a queenless colony. In addition, we used an antibody against a nucleolar marker, fibrillarin, in the colocalization experiments. Fibrillarin is a highly evolutionarily conserved family of nucleolar proteins that are associated with small nucleolar RNAs and have roles in the early processing and modification of pre-rRNA [45]. HEX 110 colocalized with fibrillarin in nucleolar regions of the terminal filament cells (Fig 5A–5D) and developing follicles (Fig 5E–5H). HEX 110/fibrillarin colocalization was evident in the several nucleoli typically found in the nurse cells and in the single nucleolus of the follicle cells involving the oocyte (at stage 2, see Fig 2) (Fig 5E–5H). Colocalization values expressed as Manders coefficients and fluorescence intensity are specified in Fig 5 legend. The images in Fig 5 clearly show that HEX 110 is associated with fibrillarin in the nucleoli of nurse and follicle cells.

**Fig 6:**
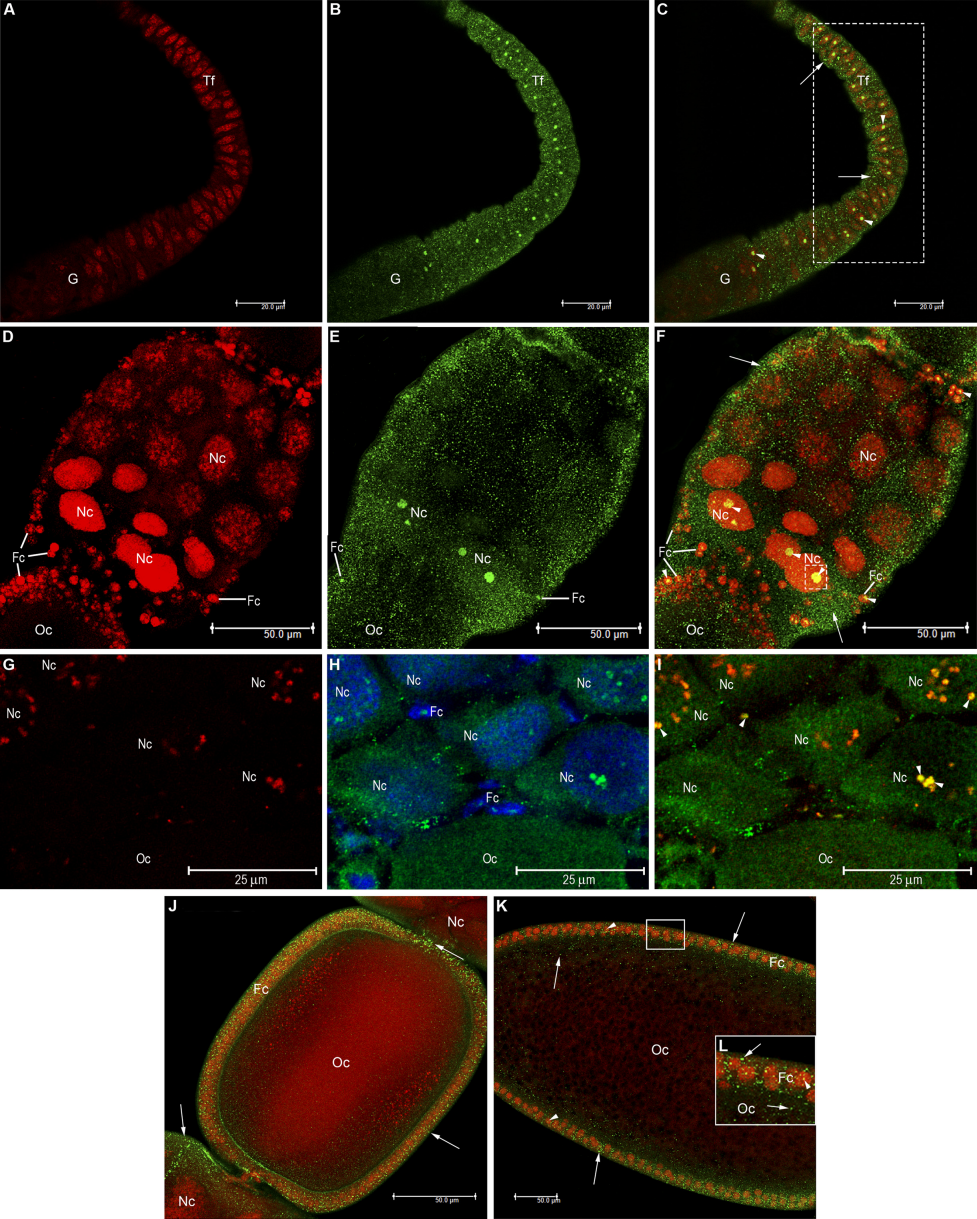
Immunolocalization of HEX 110 in the queen active ovarioles (stage S4). (A-C) Part of the terminal filament (Tf) and upper region of the germarium (G) stained with (A) propidium iodide (red), which stains RNA and DNA, or with (B) anti-HEX 110/Alexa-Fluor 488; (C) merged image. Arrowheads point to nucleolar HEX 110/propidium iodide foci (yellowish green). Arrows point to cytoplasmic HEX 110 foci (green). The area delimited by the dashed rectangle was used for estimation of Manders colocalization coefficients, M1 (red) and M2 (green), resulting in values of 0.2902 and 0.2208, respectively (red and green signals amounted to 20.33% and 16.38% of the delimited area, respectively). (D-F) Developing oocyte (Oc, stage 3–4, see Fig 2), nurse cells (Nc) in the nurse cell chamber and follicle cells (Fc). (D) propidium iodide. (E) anti-HEX 110/Alexa-Fluor 488. (F) Merged image showing yellow foci (arrowheads) indicating HEX 110/propidium iodide colocalization (and thus, HEX 110/RNA or DNA association) in the nuclei or the nurse cells (Nc) (in the basal region of the nurse cell chamber) and in the follicle cells (Fc). Arrows point to cytoplasmic HEX 110 foci. The area delimited by a dashed square was used for estimation of Manders colocalization coefficients, M1 (red) and M2 (green), resulting in values of 0.4014 and 0.2404, respectively (red and green signals amounted to 35.19% and 22.33% of the delimited area, respectively). (G-I) Magnification of part of a follicle showing nurse cells (Nc) stained with (G) anti-Fibrillarin/Alexa-Fluor 594 (red foci) or (H) anti-HEX 110/Alexa-Fluor 488 (green foci) plus DAPI (blue). The yellow foci in the cells of the merged (I) image (arrowheads) identify nucleoli and confirm their presence in the basal region of the nurse cell chamber (as seen in F image). Follicle cells (Fc) and part of an oocyte (Oc) are also visible in H and I images. (J-L) Merged HEX 110/propidium iodide-stained images. (J) Oocyte (Oc, stage 5, see Fig 2) involved by somatic epithelial follicle cells (Fc) and separated from the nurse cell chamber. HEX 110 foci (green) are mainly seen in the cytoplasm of follicle cells (Fc) and in the nurse cell (Nc) chamber (arrows). The propidium iodide red-stained oocyte (Oc) reflects its large amount of cytoplasmic RNA. (K) The quasi-mature oocyte (Oc, stage 7, see Fig 2) surrounded by follicle cells (Fc) in the lower portion of the ovariole. HEX 110 foci are evident in the cytoplasm of follicle cells (Fc) and in the peripheral cytoplasm of the oocyte (arrows). Arrowheads point to colocalized HEX 110/propidium iodide (yellow foci). (L) Magnification of part of the follicular epithelium and ooplasm. HEX 110 foci are mainly evident in the follicle cell (Fc) cytoplasm and ooplasm (arrows, green foci). In the nuclei of the follicle cells, HEX 110 clearly colocalized with propidium iodide (Fig 6L, yellow foci pointed by arrowhead).

Subsequently, we examined HEX 110 foci in the active ovaries (S4 staged ovarioles, see Fig 2) of egg-laying queens incubated with anti-HEX 110 and the nucleic acid (DNA and RNA)-labeling reagent, propidium iodide. Like observed in queenless workers (Fig 5A–5D), HEX 110 foci were localized throughout the terminal filament and upper portion of the germarium (Fig 6A–6C), as well as in the follicular region (Fig 6D–6L). HEX 110 and propidium iodide colocalized in the nucleolar region of the terminal filament cells (yellowish foci in Fig 6C) and in the nurse cells of the developing follicle (yellow foci in Fig 6F), but only in those nurse cells basally localized in the nurse cell chamber. Colocalization values (Manders coefficients and fluorescence intensity) established for Fig 6C and 6F are indicated in the respective figure legend. The small follicle cells localized around the oocyte (stage 3–4, see Fig 2) and around the nurse cell chambers also showed nuclear yellow foci indicating HEX 110/propidium iodide colocalization (Fig 6F).

In order to verify whether the large and yellow-stained nuclear structures detected in the nurse cells (Fig 6F) were in fact nucleoli, we used the nucleolar marker, fibrillarin, in colocalization experiments with anti-HEX 110. Fig 6G–6I shows a magnified portion of a follicle labeled with anti-fibrillarin (G), anti-HEX 110/DAPI (H), and the merged anti-fibrillarin/anti-HEX 110 image (I). We could clearly observe the yellow foci in the nurse cells indicating colocalization (Fig 6G), thus confirming the presence of HEX 110 in the large nucleoli of basal nurse cells.

As oogenesis progressed, the well-defined follicular epithelium surrounding a stage 5-oocyte (see Fig 2) showed cytoplasmic HEX 110 foci, which were also apparent in the peripheral ooplasm (Fig 6J). HEX 110 foci were also detected in the nurse cell chambers localized anteriorly (top) and posteriorly (bottom) to the chamber containing the stage 5-oocyte (Fig 6J). Fig 6K shows the quasi-mature oocyte (stage 7, see Fig 2) surrounded by the follicular epithelium. HEX 110 foci were detected in the cytoplasm of the follicle cells and oocyte periphery (Fig 6L). Although scarce, HEX 110 foci also colocalized with propidium iodide in the nuclear region of some follicle cells (Fig 6K and 6L) thus indicating HEX 110/RNA or DNA association. At this stage, the nurse cell chamber accompanying the oocyte is degenerating.

### HEX 110 is sensitive to RNase A treatment

**Fig 7:**
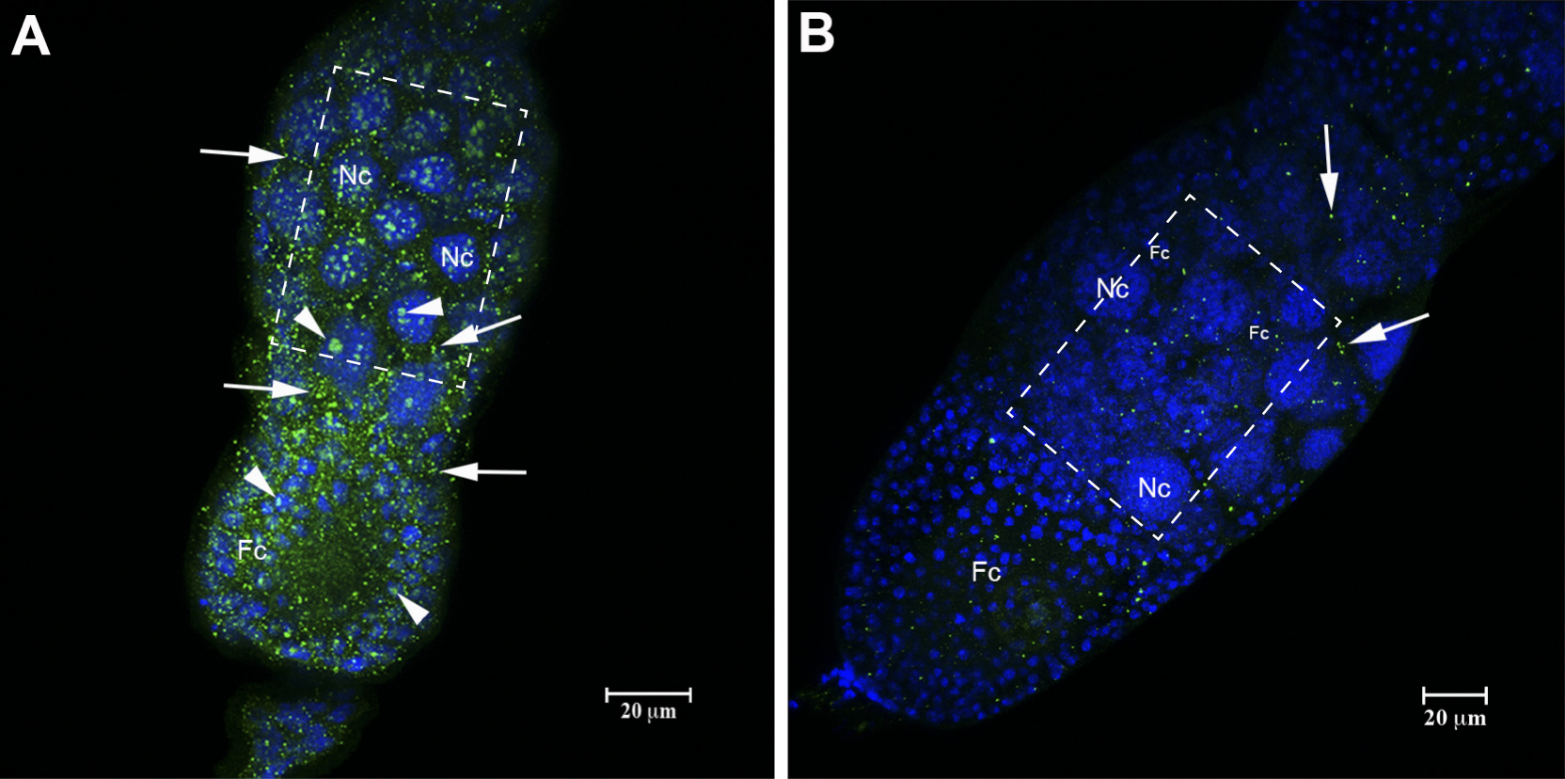
Immunolocalization of HEX 110 in RNase A-treated ovarioles (stage S2) of queenright nurse workers. (A) Control ovariole non-incubated with RNase A. HEX 110 green foci (labeled with anti-HEX 110/Alexa-Fluor 488) are abundant in the nucleus (arrowheads) and cytoplasm (arrows) of nurse (Nc) and follicle (Fc) cells surrounding the oocyte (stage 2, see Fig 2). (B) Ovariole incubated with RNase A shows scarce HEX 110 foci (arrows). DAPI-staining (blue) in B highlights the small nuclei of follicle cells (Fc) that surround the oocyte (stage 1, see Fig 2) and are interspersed between the large nurse cell (Nc) nuclei. Quantification of pixels intensity in the selected areas (dashed rectangles) of the images obtained from RNase A-treated and untreated ovarioles showed lower intensity in RNase A-treated (Mean: 17.29; Min/Max gray values: 0/255; IntDen: 575.51) than in untreated controls (Mean: 29.39; Min/Max: 0/255; IntDen: 978.45).

Treatment with RNase greatly reduced the abundance of HEX 110 foci in the ovarioles (Fig 7), thus suggesting that HEX 110 is somehow associated with cytoplasmic and nucleolar RNA in the ovariolar cells (see intensity measurements of HEX 110 pixels in Fig 7 legend). This conclusion is reinforced by the results obtained from a search of predicted RNA-binding residues in the HEX 110 protein subunit using sequence-based computational methods. Amino acid residues and regions in the HEX 110 molecule were predicted to be RNA-binding sites when we used five different predictors: RNABindR [39], BindN [36], PPRint [37], SVM [38], and RNABindRPlus [40] (S1 Fig).

### HEX 110 colocalizes with pyronin Y-labeled RNA

**Fig 8:**
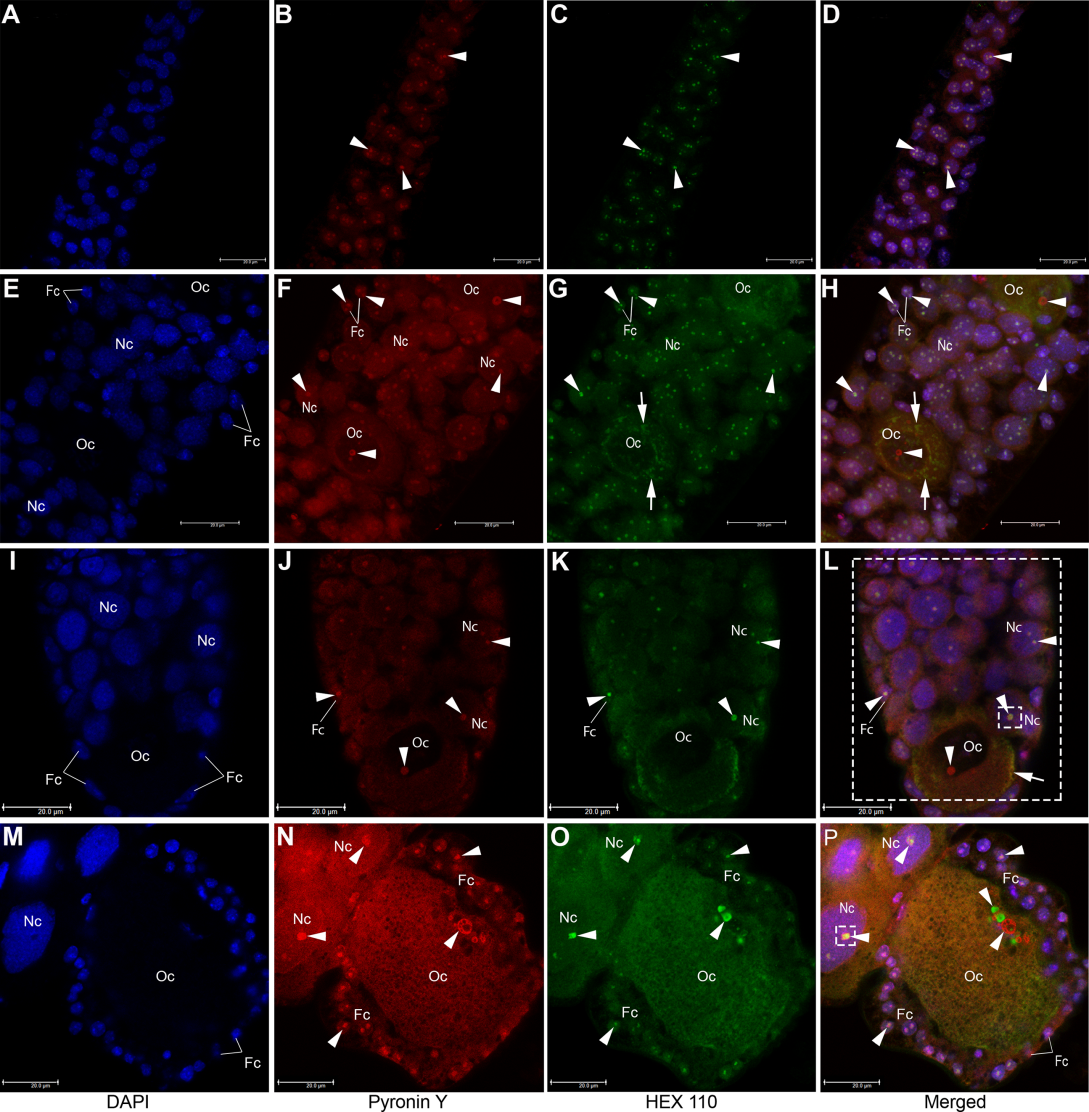
Colocalization of HEX 110 and pyronin Y, a RNA-specific stain, in the ovarioles (stage S2) of queenright nurse workers. Upper (A-D) and lower (E-H) regions of the germarium. Developing follicles immediately below the germarium (I-L) and at the botton of the ovariole (M-P). Oc: oocyte; Fc: follicle cell; Nc: nurse cell. Arrowheads and arrows show nuclear and cytoplasmic foci, respectively, identified with anti-HEX 110/Alexa-Fluor 488 (green foci) or pyronin Y (red foci). (D, H, L, P) The yellowish/yellow foci indicate HEX 110/RNA colocalization in the nucleoli of follicle (Fc) and nurse (Nc) cells, but not in the oocyte (Oc) nucleolus. (L) HEX 110 colocalizes with RNA in the periphery of the stage 1-oocyte cytoplasm (Oc, arrow). The estimated M1 (red) and M2 (green) Manders colocalization coefficients for the large area delimited by dashed lines were 0.4971 and 0.4399, respectively (red and green signals amounted to 51.59% and 44.92% of the selected large area). For the small area delimited by a dashed square, the M1 and M2 coefficients were 0.3434 and 0.2567, respectively (red and green signals amounted to 40.36% and 30.94% of the small area, respectively). (P) HEX 110 appears in the nucleus of the stage 2-oocyte (Oc) at the base of the ovariole (nuclear green foci pointed by arrowhead), but is not colocalized with RNA (nuclear red foci pointed by arrowhead). HEX 110 colocalized with RNA in the nucleoli of nurse cells (Nc): the M1 and M2 values for the nurse cell nuclear area enclosed by the dashed square corresponded to 0.2883 and 0.2624, respectively (red and green signals amounted to 35.99% and 35.11%, respectively).

Pyronin Y, a dye that intercalates and stains RNA red, was used as an additional staining method to investigate the association between HEX 110 (green foci) and RNA (red foci) in the ovariole (S2 staged-ovariole, see Fig 2). This association (revealed as yellowish/yellow foci) was evident in the nucleoli of the germline and somatic cell precursors (Fig 8A–8D), and in their derived nurse and follicle cells (Fig 8E–8P), where HEX 110 foci colocalized with pyronin Y stained RNA (see colocalization values expressed as Manders coefficients and fluorescence intensity in Fig 8 legend). However, a different labeling pattern was observed in the growing oocytes. Initial oocytes transiting from the germarium to the follicular region did not show HEX 110/RNA colocalization in the nucleolus, nucleoplasm or cytoplasm (Fig 8F–8H). Oocytes at the stage 1 (see Fig 2), like that shown in Fig 8I–8L) revealed HEX 110 foci/RNA colocalization only in the cytoplasm periphery. In the most basal oocyte (stage 2) (Fig 8M–8P), HEX 110 foci were evident in the nucleus, but completely dissociated from pyronin Y stained RNA.

### *In silico* analysis suggests active transport of HEX 110 through the nuclear envelope and supports a nucleolar localization

Using an accurate computer program for prediction of nuclear localization signals (NLSs), the cNLS Mapper tool [41], we identified a sequence of amino acid residues (PNVKQRAADQDLLNKQQDVIQLLQKISQPI) at the 18 to 47 positions in the HEX 110 protein with an NLS activity score of 4.7, meaning that the protein is localized to both the nucleus and the cytoplasm. In addition, a leucine-rich nuclear export signal (NES) comprising the residues LALVAL-V at the 8–13 and 15 positions, which may confer export activity, was predicted in the HEX 110 sequence when we used the NetNES 1.1 Server [42] (S2 Fig). Moreover, a web server for predicting protein subnuclear localization, Nuc-PLoc [43], indicated the nucleolus as the compartment for HEX 110 localization. Together, such bioinformatics analyses suggest active transport of HEX 110 through the nuclear envelope, thus agreeing with our immunofluorescence image analyses showing HEX 110 in the cytoplasm and nucleolus of ovarian cells.

## Discussion

### HEX 110 is a cytoplasmic and nucleolar protein in ovariolar cells

Using a specific antibody, HEX 110 was localized in the cytoplasm and nucleus of germline and somatic ovarian cells, thus allowing us to infer that it is transported through the nuclear envelope. It was generally agreed that only small proteins with a maximum size of 60 kDa were able to passively diffuse through nuclear pore complexes. This notion changed since it was demonstrated that proteins whose sizes were 90 to 110 kDa diffuse into the nucleus [46]. Therefore, the size does not seem to be an obstacle for the HEX 110 protein to diffuse through the nuclear pore and reach the nucleolus. And even if its size prevents a passive diffusion, the HEX 110 protein may penetrate the nucleus via active transport mediated by an NLS sequence. NLS and NES sequences were both identified in the HEX 110 protein through *in silico* analysis, thus supporting its transport between cytoplasm and nucleus in the ovarian cells.

The presence of HEX 110 in the ovarian cells, remarkably in the nucleolus, as confirmed by colocalization with fibrillarin, definitively points to novel roles in honeybee physiology in addition to being a larval storage protein. Our confocal images do not allow us to specify the exact localization of HEX 110 in the nucleolar subcompartments. Therefore, we do not know whether HEX 110 exists in the fibrillar centre or the dense fibrillar component, both representing nucleolar regions expressing fibrillarin [47, 48]. Such structural organization, as well as the exact nucleolar localization of HEX 110, may be further resolved using specific antibodies against proteins that are markers of the distinct nucleolar subcompartments.

The nucleolus is the site of the ribosomal RNA (rRNA) gene cluster and rRNA production, processing and assembly with ribosomal proteins. The nucleolus may also have roles in RNA transport, RNA modification and cell cycle regulation [49]. In the nucleolus, HEX 110 showed a clear colocalization with pyronin Y, a small fluorescent probe used to label endogenous RNA (mRNA, tRNA and rRNA). Furthermore, HEX 110 is sensitive to RNase treatment and has potential RNA-binding sites, these characteristics supporting its association with RNA. Interestingly, in the cytoplasm HEX 110 may perhaps be a partner of the coprecipitated DIS3-like exonuclease, which is annotated as an exoribonuclease in the honeybee genome assembly. The unspecific pull down may be indicative of interaction between HEX 110 and this exoribonuclease, since it is known that hexamerins may integrate protein complexes [50]. DIS3-like exonuclease sequence does not have NES or NLS signals. Thus, it is a cytoplasmic enzyme with potential roles in RNA degradation and may target RNA-protein complexes. This seems one more aspect associating HEX 110 and RNA.

HEX 110 has a nucleolar localization in the precursors of oocytes, nurse cells and follicle cells. This pattern is maintained in the differentiated nurse and follicle cells. However, in general, the growing oocytes did not show nucleolar HEX 110 foci, despite the presence of cytoplasmic foci and scarce nucleoplasmic foci (see Fig 8H). As the oocyte develops, the presence of HEX 110 foci in the nucleoplasm becomes more evident as seen in the oocytes occupying the follicles located at the most basal region of the ovarioles (see Fig 8O and 8P). In these basal oocytes, however, the nuclear HEX 110 foci are dissociated from the nuclear RNA foci. Oocytes in the basal follicles of queenright bee ovaries are arrested in the meiotic prophase [51] and tend to be eliminated by programmed cell death [52]. At this stage of knowledge on HEX 110 non-canonical function(s), a conceivable role related to these events occurring in the basal oocyte would only be mere speculation.

Proteins may change their cellular distribution in response to changes in transcription during the cell cycle or cell differentiation. Nucleolar proteins may associate with, or dissociate from, other nucleolar or nuclear proteins, thus altering their position and concentration in the sub-nuclear compartments [53]. This is also a possibility in the specific case of nuclear HEX 110 foci distribution in the nurse cells associated with each ovariolar follicle. Depending on cell type and cell physiology status, the nucleolus may change its structure and components [48]. In addition, a single cell may have more than one or even several nucleoli that may fuse to form a single larger nucleolus. Nucleolus size is indicative of the metabolic activity of the cell, and in general, large nucleoli are observed in cells that are actively synthesizing proteins [54]. We observed several small nucleoli in the nurse cells integrating the active ovaries of queenless workers (Fig 5D). The presence of multiple nucleoli in germline cells is not an uncommon occurrence, and the nurse cells derive from the same germ cell precursors generating the oocytes. As an example, thousands of nucleoli are typically found in oocytes of the amphibian experimental model, *Xenopus laevis* [54]. Such a pattern, however, changed in the fully active ovarioles of the honeybee queen, where larger nucleoli, which may result from the fusion of the small ones, were observed in the nurse cells located at the basal region of nurse cell chambers. These nurse cells are actively engaged in RNA and protein syntheses for incorporation in the growing oocyte, and their large nucleoli apparently reflect such high metabolic activity. After massive mRNA production during oogenesis, these cells reduce their metabolic activity and eventually die during the final steps of egg formation [51, 55].

Determining the localization of a given protein in the tissues or cells may provide clues to its function, or functions, and this strategy is particularly important when we are dealing with novel proteins or proteins that are suspected to exert additional functions, besides the canonical one, as HEX 110. Attempts at characterizing proteins localized to nuclear subcompartments of mammalian cell lines [53, 56] and the use of proteomics [49, 57] have improved the identification of nucleolar proteins. Importantly, the NOPdb database, version 3.0 (http://www.lamondlab.com/NOPdb3.0/), provides coverage of the human nucleolar proteome that comprises over 4500 proteins [58]. Although only a fraction of the proteins deposited in NOPdb3.0 may primarily reside in the nucleolus—the other proteins being transiently expressed in this nuclear domain [56], the database gathers important proteomics information potentially able to improve our understanding on nucleolar functions. HEX 110 is an insect protein and, unfortunately, it is not represented in the NOPdb3.0 nucleolar proteome.

### HEX 110 and ovary status

HEX 110 foci were detected in the inactive ovaries of newly emerged and nurse workers living in queenright colonies, in the activated ovaries of queenless workers and in the fully active ovaries of queens. HEX 110 foci are present from the apical to the basal ovariole length. Consequently, there are many more HEX 110 foci in the active ovarioles simply because they are longer and larger than the inactive. The truly comparable regions are the terminal filament and the uppermost portion of germarium, which are morphologically similar in the inactive and active ovarioles. We carefully examined these regions in our image bank and did not find any evidence of differences in the distribution pattern, abundance or intensity of HEX 110 foci, as monitored by immunofluorescence and confocal microscopy.

A deep sequencing approach (high-throughput RNA sequencing technology) recently revealed the transcriptomes of active and inactive ovaries of honeybee queens and workers. *hex 110* transcripts were found in the ovaries independently of their functional status (active/inactive) and of the social condition (queen/worker) [59]. By showing HEX 110 foci in the inactive and active ovaries, our confocal images agree with and validate the transcriptome data. However, the RNA-seq approach [59] revealed higher levels of *hex 110* transcripts per μg of RNA in the inactive than in the active ovaries. Similarly, using the same RNA-seq technology, unpublished data from our laboratory (L.M.F. Macedo and Z.L.P. Simões) also revealed a higher number of *hex 110* reads, which is used as a proxy of transcript quantity, in the inactive than in the active ovaries of honeybees.

Nothing is known about the stability or turnover rate of HEX 110 protein. Many regulatory mechanisms occur after the mRNA is produced; therefore, the levels of a given transcript may not necessarily reflect the abundance of the corresponding protein [60]. However, when *hex 110* transcript levels were quantified in the fat body tissue, where hexamerins are abundantly produced, we found a contrasting result. As demonstrated previously [11], a high level of *hex 110* transcripts in the honeybee fat body was associated with activated ovaries. This result, obtained by quantifying *hex 110* transcripts through RT-qPCR assays, indicates that *hex 110* expression is distinctly regulated in the fat body and ovaries.

One would even ponder whether the HEX 110 protein foci detected in the honeybee ovaries are exclusive products of ovariolar cell transcription, or whether the fat body contributes via a dynamic process of HEX 110 secretion and uptake by the ovaries. A well-studied example of a storage protein abundantly produced in the fat body, secreted into hemolymph, and sequestered by the ovaries is vitellogenin, the main protein in the egg yolk [61–64]. However, this does not seem to occur with the HEX 110 protein, considering that it is faintly detected, or not detected, in the haemolymph of adult workers [11, 18]. Thus, the HEX 110 foci found in the ovaries likely represent products of ovarian biosynthesis, but this question is still a matter for further research.

Herein we demonstrated the localization of HEX 110 in the cytoplasm and nucleolus of ovariolar cells, which is a valuable finding for elucidating functional aspects of hexamerins. Further experiments are required to clarify whether HEX 110 is a regulatory or structural protein in the ovaries and, mainly, to identify the interaction partners of the nucleolar HEX 110 protein.

## Supporting Information

S1 Fig Putative RNA-binding amino acid residues in the HEX 110 sequence. RNABindR (yellow), BindN (red), PPRint (green), SVM (pink), and RNABindRPlus (blue) were used as sequence-based computational packages. The asterisks indicate RNA-binding residues predicted by all of these computational packages. Plus signs indicate residues identified by four of these predictors.
(TIF)

S2 Fig Prediction of Nuclear Exporting Signal (NES) and Nuclear Localization Signal (NLS) in the HEX 110 sequence. (TIF)

S1 Table Alignment scores (Honeybee genome v. 4.5, NCBI BLASTP) for the polypeptide hits obtained through mass spectrometry of immunoprecipitated proteins. (PDF)
