# Supplementary figures and images for: The HEX 110 Hexamerin Is a Cytoplasmic and Nucleolar Protein in the Ovaries of Apis mellifera
Source: PLoS One. 2016 Mar 8;11(3):e0151035. doi: 10.1371/journal.pone.0151035 (PMC4783013; doi:10.1371/journal.pone.0151035)

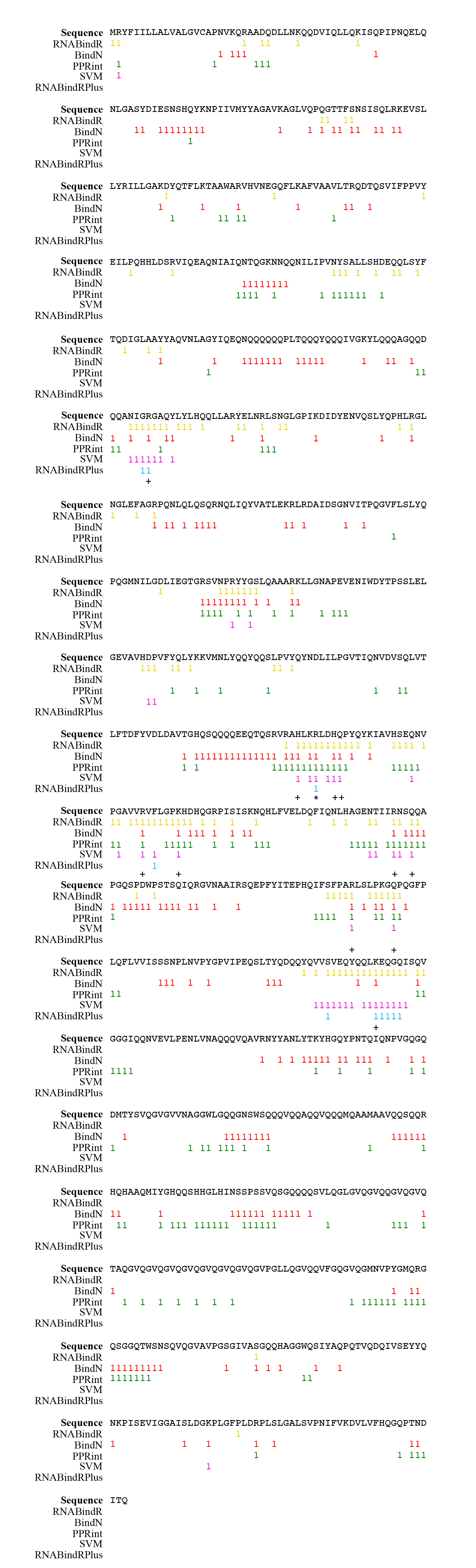

Supplement: S1 Fig — RNABindR (yellow), BindN (red), PPRint (green), SVM (pink), and RNABindRPlus (blue) were used as sequence-based computational packages. The asterisks indicate RNA-binding residues predicted by all of these computational packages. Plus signs indicate residues identified by four of these predictors. (TIF) [file pone.0151035.s001.tif]

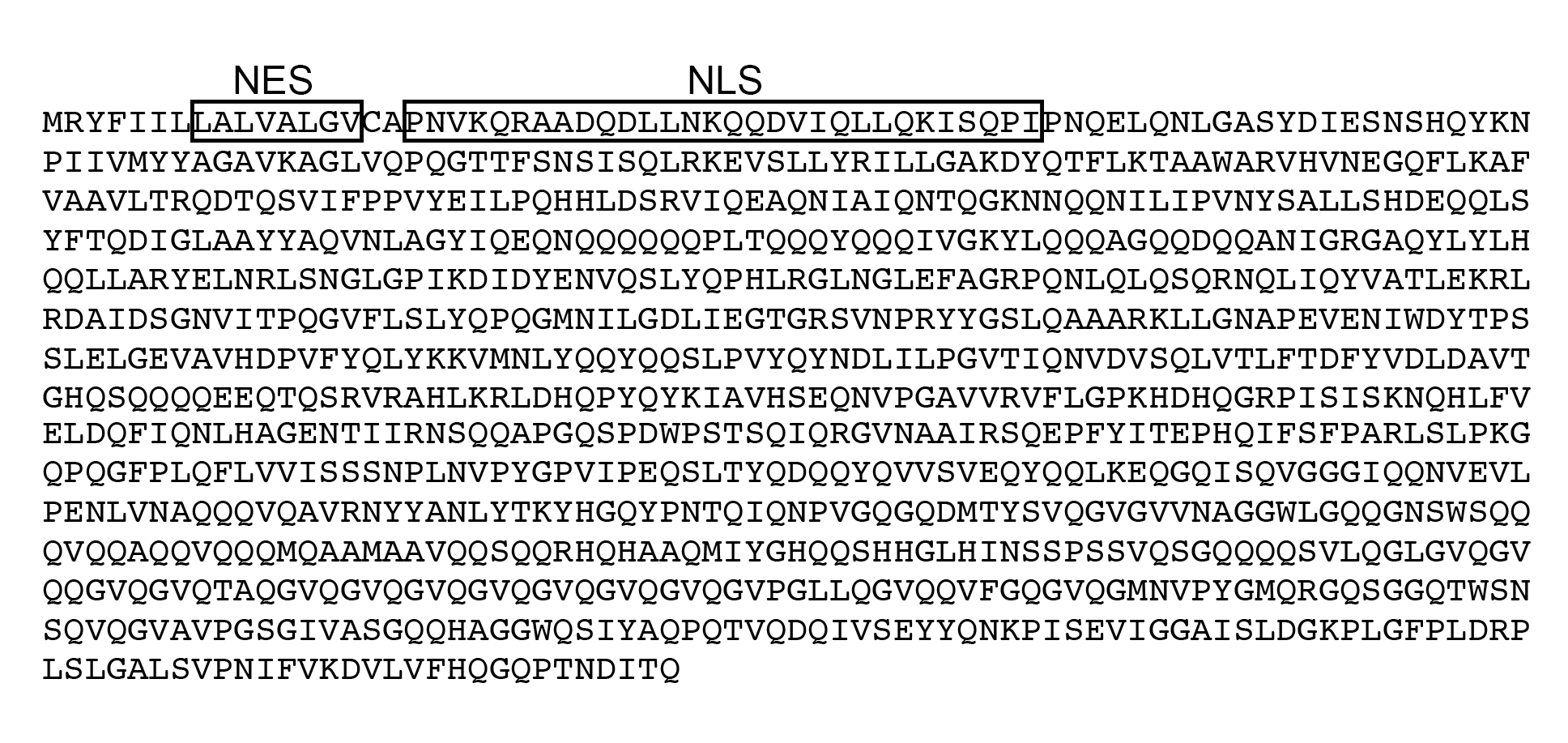

Supplement: S2 Fig — (TIF) [file pone.0151035.s002.tif]
