# Supplementary material for: The HEX 110 Hexamerin Is a Cytoplasmic and Nucleolar Protein in the Ovaries of Apis mellifera
Source: PLoS One. 2016 Mar 8;11(3):e0151035. doi: 10.1371/journal.pone.0151035 (PMC4783013; doi:10.1371/journal.pone.0151035)
Supplement: S1 Table — (PDF) [file pone.0151035.s003.pdf]

Protein	Peaks	Max score	Total score	Query cover	E-value	Identity
Hexamerin	1	56.2	70.8	100%	5e-11	100%
	2	63.4	237	100%	3e-13	100%
	3	66.8	98.6	100%	3e-14	100%
	4	73.6	109	100%	1e-16	100%
	5	73.6	73.6	100%	1e-16	100%
DIS3-like exonuclease	6	55.4	71.7	90%	2e-10	80%
